# Editorial: Host-bacteria interactions in fish pathogens

**DOI:** 10.3389/fcimb.2024.1515641

**Published:** 2024-11-14

**Authors:** Jose Ramos-Vivas, Félix Acosta

**Affiliations:** ^1^ Research Group on Foods, Nutritional Biochemistry and Health, Universidad Europea del Atlántico, Santander, Spain; ^2^ Research Group on Foods, Nutritional Biochemistry and Health, Universidad Internacional Iberoamericana, Campeche, Mexico; ^3^ Grupo de Investigación en Acuicultura (GIA), Instituto Ecoaqua, Universidad de Las Palmas de Gran Canaria, Las Palmas de Gran Canaria, Spain

**Keywords:** host-pathogen, bacterial virulence, fish pathogen, fish disease, cellular microbiology, fish immune response

In order to promote the sustainable development of aquaculture, it is of great importance to better understand fish diseases caused by classic and emerging bacterial pathogens. Strains of classic fish pathogens such as Aeromonas, Vibrio, Photobacterium, Edwardsiella, Yersinia, Flavobacterium, or Piscirickettsia. Studies of these fish diseases should focus on better understanding the basis of the interaction between pathogens and their host, both at a cellular and molecular level, with the aim of creating new tools to combat them. While Cellular Microbiology has grown exponentially in the study of animal and human bacterial pathogens, the study of the interaction of fish bacterial pathogens with cells has made virtually no progress. This is partly due to the lack of well-characterized fish cell lines, and poor homogeneity in studies with infection models. In this Research Topic in Frontiers in Cellular and Infection Microbiology, encouraged collaborations between bacteriologists, veterinarians, immunologists, and cell biologists. This Research Topic of Frontiers in Cellular and Infection Microbiology brings together cutting-edge research that sheds light on various aspects of these interactions, from molecular mechanisms to potential therapeutic strategies.

## Comprehensive analysis of the JAK-STAT pathway

The papers we have published certainly leave us wanting more, as many other bacterial fish pathogens can offer researchers new avenues of research, but also on the part of their aquatic hosts. This can be clearly observed in the paper by Rao et al. demonstrating the important role of the Janus kinase/signal transducers and activators of transcription (JAK-STAT) as a key players in fish immunity. Using the lumpfish (Cyclopterus lumpus L.) as a model, Rao et al. characterized seven STAT genes and conducted a comprehensive analysis of the JAK-STAT pathway components after challenge with the fish pathogen Vibrio anguillarum. Their findings highlight the conservation of STAT genes among fish species and also demonstrate the distinct activation patterns of the JAK-STAT pathway in response to different types of pathogens (i.e., the response against poly I:C, which mimics a viral infection was very different). The implications of this research extend beyond lumpfish, providing solid foundation for future studies on the functional significance of these genes in various fish species. Also, this knowledge can help in the development of targeted vaccination strategies, addressing the pressing need for prophylactic measures against both bacterial and viral diseases in aquaculture.

## Bacterial co-infections: a complex interplay

While single-pathogen infections have been the focus of much research, the reality in aquatic environments is often more complex. A study on Piscirickettsia salmonis, the causative agent of Salmon Rickettsial Septicemia (SRS), has shed light on the intricacies of bacterial co-infections (Carril et al.). This facultative intracellular bacterium is known to exist in two genogroups (LF-89 and EM-90) with varying virulence levels. The researchers investigated the effects of co-culturing these genogroups both *in vitro* and *in vivo* in Atlantic salmon (Salmo salar). The results of this study were eye-opening. *In vitro* experiments revealed that co-cultures of strains LF-89 and EM-90 exhibited growth patterns similar to EM-90 monocultures, with a higher ratio of EM-90 over LF-89 throughout the co-culture period. This competitive interaction was further elucidated through the examination of virulence factors expression. The luxR gene, for instance, was expressed only in EM-90-like isolates, while significant differences in flaA and cheA expression were observed between mono- and co-cultures. Perhaps, the most intriguing findings came from the *in vivo* co-culture experiments. Transcriptomic analysis revealed an upregulation of transposases, flagellum-related genes (fliI and flgK), transporters, and permeases in these experiments. These changes could potentially unveil novel virulence effectors employed by P. salmonis during the early stages of infection. The implications of this research are far-reaching. It demonstrates that the cohabitation of different genogroups of a pathogen can modulate their behavior and virulence factor expression. This knowledge is crucial for developing more effective treatment strategies against complex infections in salmonid aquaculture.

## The role of bacterial appendages in virulence

Bacterial surface structures play a vital role in pathogenesis, and recent studies have provided new insights into their functions in fish pathogens. One such study in the Research Topic focused on the tight adherence (Tad) pili, a unique Type IV class C pilus in virulent Aeromonas hydrophila (vAh) [3]. Through a comprehensive comparative genomics analysis of 170 A. hydrophila genomes, the researchers confirmed the conserved presence of the Tad operon in vAh isolates, suggesting its potential contribution to pathogenicity. To elucidate the specific role of Tad pili in A. hydrophila virulence, the entire Tad operon was knocked out in the ML09-119 strain in the work of Tekedar et al. The results were striking: while the absence of the Tad operon did not affect growth kinetics, it significantly reduced virulence in catfish fingerlings and decreased biofilm formation. Interestingly, the Tad mutant exhibited increased sensitivity to low pH conditions but showed no changes in sensitivity to other environmental stressors. These findings highlight the multifaceted role of Tad pili in vAh pathogenesis and biofilm formation, underscoring the importance of Type IV pili in bacterial infections of fish.

## Emerging pathogens: a case study of Vibrio alginolyticus

As aquaculture continues to expand globally, the emergence of new pathogens poses significant challenges to the industry. A recent study on Vibrio alginolyticus SWS, isolated from a disease outbreak in Scylla serrata (mud crab) aquaculture in Hong Kong, provides valuable insights into the characteristics and pathogenicity of this emerging pathogen. The team lead by Dr. Seto employed a multifaceted approach, including experimental infection, histopathology, whole genome analysis, and biochemical characterization, to investigate the impact of V. alginolyticus SWS on S. serrata K (Ka Kwok et al.). This pathogen can cause up to 70% mortality during summer outbreaks and 75% mortality in experimental infections. We are pleased that crustaceans have been included in this Research Topic. Although our first idea was to learn more about pathogen interactions with fish, non-classical models help us to add information on the virulence of some classic and emerging fish pathogens, but which do not only cause infections in fish. In this case, there are also no crustacean cell lines that have been frequently used to study the virulence of these pathogens. Infected mud crabs exhibited a range of symptoms, including inactivity, loss of appetite, and tissue discoloration. Histopathological analysis revealed significant damage to the hepatopancreas, gills, and claw muscle, indicating both direct and indirect impacts of the infection.

This comprehensive characterization of V. alginolyticus SWS as an emerging pathogen in S. serrata aquaculture underscores the need for ongoing surveillance and early detection strategies. It also highlights the importance of developing targeted disease management approaches to mitigate the economic impact of vibriosis outbreaks in mud crab farming.

From a cellular microbiology perspective, we encourage researchers to establish cell lines that allow for easier experiments to observe *in vitro* the virulence characteristics of V. alginolyticus in these models.

## The multifaceted role of RNA chaperones in bacterial pathogenesis

Moving beyond structural components and classic virulence factors such as flagella, recent research has shed light on the importance of regulatory elements in bacterial pathogenesis. A study on Edwardsiella ictaluri, the causative agent of enteric septicemia in catfish (ESC), has revealed the crucial role of the RNA chaperone Hfq in various aspects of bacterial physiology and virulence [5]. In pathogens, sRNAs and their chaperones exert broad impacts on both bacterial physiology and virulence, highlighting the central role of these systems in bacterial pathogenesis.

By creating an hfq mutant (EiΔhfq) through in-frame gene deletion, Akgul et al. were able to elucidate the multifaceted role of this protein in E. ictaluri (Akgul et al.). The deletion of hfq resulted in reduced growth rate, diminished biofilm formation capacity, and increased motility. Moreover, EiΔhfq showed impaired growth under acidic and oxidative stress conditions, highlighting the importance of Hfq both in virulence and in stress response mechanisms.

Perhaps most significantly, the hfq mutant exhibited reduced survival within catfish peritoneal macrophages and demonstrated attenuated virulence *in vivo*. These findings underscore the pivotal role of hfq in E. ictaluri, affecting its growth, motility, biofilm formation, stress response, and virulence both in macrophages and within the catfish host. Authors used a simple model to measure the survival of bioluminescent E. ictaluri cells in peritoneal macrophages obtained from channel catfish, in the presence of complete or inactivated serum. Since a prerequisite for internalization and intracellular survival of the bacteria is adherence to cell surfaces, the authors have used another *in vitro* model of epithelial cells. The deletion of hfq resulted in increased E. ictaluri killing by macrophages, but did not affect bacterial attachment or invasion. We encourage the authors to further develop this adherence model, which will allow us to better understand the pathogenesis of E. ictaluri in epithelial cells using other virulent or non-virulent strains.

## Host recognition of bacterial flagella: insights from shrimp

Another article on a classic virulence factor, such as the flagellum, helps us to improve our understanding of the virulence mechanisms of Vibrio alginolyticus on Litopenaeus vannamei (Pacific white shrimp) (Zhang et al.). The researchers created a V. alginolyticus mutant with multiple flagella and compared the gene expression in shrimp hepatopancreas infected with this strain, to those infected with the wild-type strain. Transcriptome analysis of shrimp revealed the upregulation of several immune-related genes in response to the hyper-flagellated strain. Of particular interest was the identification of two C-type lectins (CTLs) - galactose-specific lectin nattectin and macrophage mannose receptor 1 - that appear to be involved in flagella recognition. Furthermore, the study found that the TNF receptor-associated factor (TRAF) 6 gene was upregulated in the hepatopancreas, suggesting activation of the immune system through the TRAF6 pathway upon flagella detection by CTLs. The interaction between Vibrio alginolyticus and shrimp tissues has revealed a complex signaling pathway in response to the components of the bacterium’s flagellum, notably in terms of the immune response. These findings offer new perspectives on the molecular mechanisms underlying shrimp immune responses to bacterial pathogens. Such knowledge could be instrumental in developing novel strategies for enhancing disease resistance in crustacean aquaculture.

## Probiotic approaches to disease management

As the aquaculture industry continues to face challenges from bacterial pathogens, there is growing interest in alternative approaches to disease management, especially with regard to the use of antibiotics. The work of Macias et al. investigates the inhibition of A. hydrophila by probiotic strains of Bacillus (Macias et al.). Different strains of Bacillus isolated not only from aquatic environments, but also from other environments, have been used to determine how they are able to inhibit fish pathogens. Some recent reviews echo this research.

The research conducted by Macias et al. focused both *in vitro* and *in vivo* experiments to assess the efficacy of Bacillus strains. *In vitro* studies revealed that A. hydrophila produced β-hemolysin and metalloprotease enzymes throughout its exponential growth phase, providing insights into its virulence mechanisms. Tilapia fed with diets supplemented with different Bacillus strains showed significantly lower mortality rates when challenged with A. hydrophila compared to the control group. Moreover, the probiotic-fed fish exhibited improved weight gain, with increases ranging from 36% to 67% compared to the control group’s 30%. These findings suggest that functional diets formulated with high levels of soybean meal and supplemented with specific Bacillus strains could offer a viable alternative for protecting Nile tilapia cultures from A. hydrophila infections while simultaneously enhancing fish growth performance. This approach aligns with the growing trend towards more sustainable and environmentally friendly aquaculture practices. Probiotics may act as barriers to pathogens and toxins by preventing their adhesion to epithelial receptors. That is why adherence to the host is a classical selection criterion for potential probiotic bacteria that could result in a transient colonization to promote immunomodulatory effects.

Many studies focus on antagonism mediated by antimicrobial molecules produced by Bacillus to draw conclusions about its suitability to be a probiotic candidate, but we believe that in the field of interactions between bacteria and cells, many *in vitro* experiments are missing to be able to categorize candidates for probiotic strains as true probiotics. After all, permanence in host tissues depends on the ability of these strains to attach to cells. We believe that many types of *in vitro* experiments can be carried out to advance this aspect. We encourage authors to conduct such trials with these strains of Bacillus, which actually have probiotic potential, following the results of experimental infections.

## Conclusion and future directions

The studies presented in this Research Topic collectively paint a vivid picture of the complex interactions between fish pathogens and their hosts. More than half a dozen pathogens have been featured in this publication, some classic but others actually emerging. As we look to the future, several key areas emerge as good options for research:

1. Molecular mechanisms of host-pathogen interactions: Continued research into the molecular basis of pathogen recognition, immune activation, and bacterial virulence will be crucial for developing targeted interventions.
2. Emerging pathogens: Ongoing surveillance and characterization of new and emerging pathogens will be critical for anticipating and mitigating future disease outbreaks. In these pathogens, we must test classic assays *in vitro* and *in vivo*, but also design entirely new ones, such as the development and study of infection in crustacean cell lines. The sparse development of these tools has hindered progress in understanding fish and shellfish-pathogen interactions at the cellular level. It is clear that interdisciplinary approaches combining genomics, transcriptomics, cellular microbiology, and immunology will be key to addressing the challenges facing the aquaculture industry.

